# A Novel Algorithm to Identify Predictors of Treatment Response: Tapentadol Monotherapy or Tapentadol/Pregabalin Combination Therapy in Chronic Low Back Pain?

**DOI:** 10.3389/fneur.2019.00979

**Published:** 2019-09-13

**Authors:** Jan C. Otto, Julia Forstenpointner, Juliane Sachau, Philipp Hüllemann, Martin Hukauf, Thomas Keller, Janne Gierthmühlen, Ralf Baron

**Affiliations:** ^1^Division of Neurological Pain Research and Therapy, Department of Neurology, University Hospital Schleswig-Holstein, Kiel, Germany; ^2^StatConsult GmbH, Magdeburg, Germany

**Keywords:** chronic low back pain, pharmacotherapy, retrospective analysis, tapentadol, treatment predictors

## Abstract

**Purpose:** High dose monotherapies or drug combinations are used to achieve sufficient analgesia for the treatment of severe chronic low back pain, before invasive therapy options are considered. In order to demonstrate an alternative for an empirical treatment approach, the authors' primary aim was to present an algorithm for the objective identification of treatment predictors. Additionally, the study identified baseline-characteristics in chronic low back pain patients prior to tapentadol PR treatment, as well as scrutinized those patients, either benefitting from a medium/high dose tapentadol PR monotherapy or a combination therapy (medium dose tapentadol PR + pregabalin).

**Patients and Methods:** The statistical approach included data of a previously published randomized, double blind, phase 3b study which compared the effectiveness and safety of tapentadol PR vs. a combination of tapentadol PR and pregabalin. In total, 46 clinical parameters were included in the statistical prediction models which were applied separately either to 50 patients who already responded well during the titration period (i.e., medium dose tapentadol PR) or to 261 patients with in the comparative treatment period [i.e., monotherapy (high dose tapentadol PR) or combination therapy (medium dose tapentadol PR/pregabalin)].

**Results:** The first statistical model identified three co-variables (NRS-3, PDQ, SQ) with predictive effects on patients responding well (“optimal responders”) to a medium dose tapentadol PR titration. Those patients presented low baseline pain intensity scores, good sleep quality and high painDETECT scores. The second statistical model identified eight co-variables (PDQ, numbness, SF-12 MCS, SF-12 PCS, VAS, HADS-A, HADS-D, SQ) with predictive effects on patients responding to high dose tapentadol PR monotherapy vs. a combination therapy (tapentadol PR + pregabalin). The high dose tapentadol PR responders indicated high painDETECT scores, little numbness and a good mental health status. Whereas, the combination therapy (tapentadol PR + pregabalin) responders were characterized by severe sleep disturbances and little anxiety.

**Conclusion:** The statistical analysis characterized chronic low back pain patients and identified factors contributing to a treatment response. Thus, this retrospective statistical algorithm represents an elegant method, which may contribute to future strategies toward a more individualized and improved mechanism based pain therapy.

## Introduction

**Figure 1:**
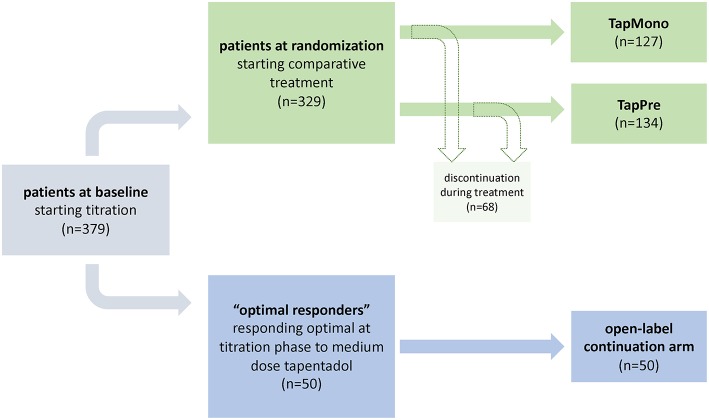
Study design. The flowchart indicates the study design of the initial trial as well as the distribution of patients treated in the open-label arm and patients which were randomized to tapentadol PR monotherapy [= TapMono] or the combination therapy tapentadol PR + pregabalin [= TapPre]. The blue path indicates the selected parameters within the first model i.e., the prediction of optimal tapentadol responders. The green path indicates the selected parameters within the second model i.e., the prediction of outcome in two arms.

The management of patients with chronic low back pain remains challenging (1–4). Therefore, if conservative non-drug treatment options (educational, psychological, and physical) are not able to reach sufficient pain relief, a multidisciplinary therapy approach should be favored. A responsible pharmacotherapy can be part of the conservative treatment steps before invasive therapy options (i.e., spinal injections, neuromodulation techniques) are weighed. Prior to the application of the pharmacotherapy, benefits and risks of the medications must be considered, as well as country specific differences with respect to the reimbursement processes. In addition to this, it is of great importance to record preexisting conditions and social history of the patients to diminish the risks of side effects and substance abuse (5). Indeed, sometimes small doses of pain medication produce sufficient analgesia with acceptable side effects (6–8). Whereas, in many other cases, high doses of strong analgesics or a combination of drugs are required (9–12). Combinations of pharmaceutical agents might be necessary to address nociceptive and neuropathic components of chronic low back pain (13, 14). Therefore, in response to mostly empirical driven treatment selections, the authors present an alternative algorithm to identify predictors of treatment response. The following analysis focuses on the clinical situation in which a patient already received a strong analgesic, in this case tapentadol PR, and a clinician has to decide if an escalation of the medication or a combination of two different agents is more appropriate. The statistical approach included data of a recent randomized, double blind trial (NCT01352741), which assessed the efficacy and safety of either a high dose tapentadol PR (prolonged release) or a combination of medium doses of tapentadol PR and pregabalin in chronic low back pain patients with neuropathic components (15). The original trial indicated similar efficacy of both treatment regimens but side effects were more frequent in the combination arm. The most frequent reported side effects were: hyperhidrosis, dizziness, nausea, somnolence/fatigue, constipations, headache, and vomiting for the tapentadol PR treatment arm; and: dizziness, somnolence/fatigue, nausea, headache, and hyperhidrosis for the tapentadol PR/pregabalin combination arm. Furthermore, the original study followed up medium dose tapentadol PR responders (i.e., “optimal responders”) with a very good pain reduction (NRS-3 <4). Those patients were monitored in a third open label continuation arm under a constant dose, after the titration phase (16) (see Figure 1).

Consequently, this study applied two different examples for *post hoc* stratification approaches. Depending on the scale of the respective outcome variable (1) binary scaled (in “optimal responders”; yes/no) or (2) continuously scaled and quasi-continuously scaled (in high dose tapendadol PR monotherapy [= TapMono] vs. tapentadol PR + pregabalin combination therapy [= TapPre]; quality of life [QoL] response variables) different linear and logistic models were applied, respectively.

The outcome of such approaches should guide the clinician in choosing the appropriate treatment strategy. In fact, for daily clinical routine, an a priori identification of patients who benefit from a medium dose medication (tapentadol PR 300 mg/day), or those patients either requiring a high dose monotherapy (tapentadol PR 500 mg/day) or a combination therapy of the investigated agents, would be a crucial step toward an individualized pain therapy (17–19). Easy to use decision tools or landmarks are necessary especially in determining a high dose or combination therapy. Thereby, the outcome of such an approach might also foster shared decision-making between patients and physicians, and in light of shrinking health resources, the careful evaluation of the risks and benefits of pain management is a prerequisite. Consequently, the proposed algorithm should help to successfully implement the individualization of pain therapy.

1. The primary aim was to present a retrospective algorithm, in order to identify patient characteristics toward treatment response in different treatment phases.
2. The secondary aims were to include data of a previously performed study (15) in order to
  - Identify baseline characteristics (prior to treatment) of patients responding very well to medium doses of tapentadol PR.
  - Identify patients who benefit from high dose tapentadol PR monotherapy or who respond better to a combination therapy of medium dose tapentadol PR and pregabalin.

## Methods

This study performed a *post hoc* analysis of the previously published randomized, double-blind, phase 3b trial (NCT01352741) (15). The original trial titrated patients to tapentadol PR (300 mg/day) over 3 weeks. Patients presenting a mild response to tapentadol PR indicated by a ≥1-point decrease in pain intensity and average pain intensity ≥4 were randomized to tapentadol PR (500 mg/day) [= TapMono] or a combination therapy of tapentadol PR (300 mg/day) + pregabalin (300 mg/day) [ = TapPre] during an 8 week comparative period. Patients reporting a dramatic pain reduction during the titration phase (NRS-3 <4, so-called “optimal responders”) were not randomized to receive additional treatment but followed up in a third open-label continuation arm under a constant dose of tapentadol PR (300 mg) (16) (see Figure 1). For further details on the study design or the assessed co-variables (i.e., questionnaires, demographic data, etc.) the authors would like to refer to the original publications (15, 16). The study was conducted in accordance with applicable local laws, the principles of the Declaration of Helsinki, and Good Clinical Practice guidelines. All patients signed an informed consent document prior to enrolling in the study. The protocol, patient information sheet, informed consent documents, and amendments were reviewed by independent ethics committees.

### Co-variables Assessed at Baseline or at Randomization

Clinical characteristics of the patients available at baseline visit (prior to treatment; baseline co-variables) or at randomization visit (randomization co-variables) were analyzed separately for prediction of treatment response to tapentadol. Beside demographic parameters (e.g., gender, BMI, medical history, vital signs, and physical examination), the focus was set on patients' self-reported characteristics (e.g., psychosocial functioning) and symptoms (e.g., sleep disruption, neuropathic pain symptoms). The following questionnaires were used to assess these parameters: pain intensity score (11 point NRS-3, average pain intensity score during the last 3 days; 0 indicates no pain and 10 indicates the worst imaginable pain), Sleep Evaluation Questionnaire (SQ), Short Form 12 Health Survey (SF-12) (20), the subject's satisfaction with treatment, EuroQol-Health State Today (EQ-5D VAS) (21), Hospital Anxiety and Depression Scale (HADS, including subscores for anxiety [ = HADS-A] and depression [ = HADS-D]) (22), patient global impression of change (PGIC), clinician global impression of change (CGIC) and the painDETECT questionnaire (PDQ) (23). The PDQ includes seven separate questions addressing specific neuropathic symptoms, i.e., burning, parenthesis, mechanical allodynia, painful attacks, thermal hypersensitivity, numbness, and pressure-evoked pain.

### Patient Selection for *post hoc* Analysis

The present *post hoc* analysis is based on patients of ≥18 years of age, with chronic low back pain lasting ≥3 months with an average pain intensity score of ≥6 on the NRS-3 at baseline. Additionally, an “unclear” or “positive” evaluation for neuropathic pain components in the PDQ questionnaire was necessary. Furthermore, all patients included were classified as suitable to receive strong analgesics (highly potent opioids) according to the guidelines set by the WHO (World Health Organization) for step III.

### Outcome Variables

In a recent study assessing the treatment effect of tapentadol in patients with low back pain, alternative patient-reported outcome measures (quality of life, functionality) were shown to be more appropriate as response criteria in prediction models than pain intensity measures (24). Therefore, the effect of co-variables on these two continuously scaled outcome variables was investigated in linear models. The QoL-response was defined as change in quality-of-life parameters derived from the SF-12 MCS sub-score (QoL-MCS-response) as well as from the EQ-5D questionnaire (QoL-EQ-5D-response). The functionality-response was defined as a change in parameters of bodily functioning derived from the SF-12 PCS sub-score (Function-PCS-response). Both outcome variables were analyzed between randomization and endpoint (8 weeks).

Furthermore, “optimal responders” were examined in relation to possible influencing and predicting base-line factors (16). Due to the binary nature of the outcome (optimal responder yes/no), predictions were analyzed in a separate logistic model.

### Statistical Prediction Models

The statistical prediction analysis was performed according to the TRIPOD statement and to recently published guidelines (25). In this study, retrospective data with a predetermined sample size were used, therefore a formal power analysis was not applicable. However, according to Harrell, prediction models should contain a number of variables <1/20 of the number of cases (26). In this case, approximately 130 datasets were analyzed, therefore the respective models should not contain more than six variables. Although this rule has been fulfilled for the final models, the full spectrum of predictive variables was used regarding variable selection and development of the models. In the following, several steps have been used to overcome this limitation:

#### Selection of Predictors

In the first step, the influence of a single variable at baseline on the outcome in bivariable models (including the baseline value of the respective outcome variable) was evaluated, in order to identify possible predictors. Depending on the scale of the respective outcome variable, continuously/quasi-continuously scaled (QoL response variables) or binary scaled (optimal responder yes/no) linear and logistic models were applied, respectively. All possible relationships were mapped for evaluation of the validity of consecutively applied models (linear relationship, monotonic relationship). In parallel, a factor analysis was performed to identify variables with a high potential for collinearity. Among strongly correlating variables, the most important (largest factor-loading) ones were selected for further analysis. As a result, a set of potential predictors was identified and included in a multivariable regression (linear or logistic regression in dependence on outcome variable, step two). Three selection processes (forward, backward, Lasso [only applicable for linear regression]) were applied. Predictors that were selected consistently [at least weakly significant (*p* < 0.05) in two out of three selection processes and at least highly significant (*p* < 0.001) in one] were used for consecutive analyses.

#### Characterization of Models and Validation

After establishing the set of possible predictors, the following analyses were performed to characterize and validate the models and to prevent overfitting. The adjusted coefficient of determination (${\text{R}}_{\text{adj}}^{2}$ indicating the part of variability of the outcome which is explained by the prediction; linear models) as well as the c-statistics (= area under ROC curve; logistic models) were used to characterize the models. Models with low values for the respective parameter (${\text{R}}_{\text{adj}}^{2}$ <0.3, c-statistic <0.6) were regarded as irrelevant. Note that c-statistic = 0.5 and ${\text{R}}_{\text{adj}}^{2}$ = 0 refer to missing as well as c-statistic = 1 and ${\text{R}}_{\text{adj}}^{2}$ = 1 to perfect prediction, respectively. In parallel, the models were reinvestigated by including the individual co-variables, from the most to the less influential. The F-change-test (linear models) as well as the likelihood test (logistic models) were used to characterize variables, which significantly improved the prediction. The robustness of the models was tested excluding aberrant ranges of values by checking whether ${\text{R}}_{\text{adj}}^{2}$ and c-statistics would considerably decrease (model is not robust) or remain stable (model is robust). This was performed by visualizing the relationship of outcome parameters and results of the model-function (predicted values). These parameters were also included in the model's estimation of the optimism by applying a previously published procedure (27). The whole selection process was applied on resampled datasets (via bootstrapping), and the gained models were applied on the original data. The difference between the related ${\text{R}}_{\text{adj}}^{2}$ and c-statistic characterizes the potential for overfitting (optimism). Consequently, the adjusted ${\text{R}}_{\text{adj},\text{corr}}^{2}$ and c-statistic_corr_ formed the values corrected by optimism.

All analyses were performed using SAS 9.2 (SAS Institute Inc., Car, NY, USA). The nomograms were calculated via R V. 3.01 software (28).

## Results

### Patient Cohorts and Prediction Models

For the prediction models 50 patients responding well to a medium dose of tapentadol PR (“optimal responders”) and who qualified for the open-label arm during the titration period (baseline until randomization, see flowchart Figure 1) were analyzed. In addition, 261 patients entering the comparative period, i.e., randomization until endpoint (127 tapentadol PR monotherapy [= TapMono]; 134 tapentadol PR + pregabalin [ = TapPre]) were included.

### Baseline Co-variables With Predictive Effect on “Optimal Responders”

For the outcome of the “optimal responders” during the titration phase three significant baseline co-variables with predictive potential were identified:

- Pain intensity score (NRS-3)
- painDETECT score (PDQ)
- Sleep Evaluation Questionnaire (SQ).

### Randomization Co-variables With Predictive Effect on Outcome Variables at Endpoint

For the outcome after randomization, the multivariable analysis revealed that eight co-variables showed significant associations with the outcome variables “quality-of-life and functionality”:

- painDETECT score (PDQ),
- painDETECT sub-score: numbness,
- Short Form 12 Health Survey, mental component summary scale (SF-12 MCS),
- Short Form 12 Health Survey, physical component summary scale (SF-12 PCS),
- EuroQol-5D-Health today overall (VAS),
- Hospital Anxiety and Depression Scale (HADS-A),
- Hospital Anxiety and Depression Scale (HADS-D),
- Sleep Evaluation Questionnaire (SQ).

### Prediction Models

#### Demographic Data as Co-variables

In all prediction models, demographic data (age, BMI, vital signs) were not associated with the treatment response.

#### Prediction Models of Optimal Responders Qualifying for the Open-Label Continuation

In the prediction model of the optimal responders (NRS-3 <4, *n* = 50), the baseline pain intensity score (NRS-3), the painDETECT score (PDQ) and the sleep quality score (SQ) had significant effects [*p* ≤ 0.05, AUC of related ROC curve: 0.67 (95% CI: 59–0.74)]. In other words, the pain intensity score should be low and sleep quality good whereas the PDQ should be high, to predict a high response (Table 1). Additionally, the Clinician Global Impression of Change score (CGIC) indicated a trend toward a predictive effect.

**Table 1:** Co-variables with predicting effect on the different treatment arms.

**Treatment arm**		**Most relevant co-variables predicting response**		
Open-label arm “Optimal responder” (tapentadol PR 300 mg/day)	Baseline co-variables	High painDETECT score	Good sleep quality	(Low NRS-3 pain intensity)
TapMono treatment arm (tapentadol PR 500 mg/day)	Co-variables at randomization	High painDETECT score	Low painDETECT subscore numbness	Good mental healt status (SF-12 MCS)
TapPreg treatment arm (tapentadol PR 300 mg/day + pregabalin 300 mg/day)		Severe sleep disturbances	Low HADS-A score	Low HADS-D score

The following equation was estimated:

$$\begin{array}{l} Optimal\;Responders=2.16-\left(0.28*CGIC\right)-\left(0.31*NRS3\right)\\ \;+\left(0.06*PDQ_{Score}\right)-\left(0.41*SQ_{Score}\right) \end{array}$$

#### Prediction Models of Different Treatment Arms

Prediction models for response were analyzed for the two different treatment arms separately (tapentadol PR monotherapy [= TapMono]; tapentadol PR/pregabalin [ = TapPre]). The aim was to identify patient characteristics at randomization which predict the response in different treatment regimes. The three models (response in MCS, PCS, EQ-5D) were used for calculation.

The number of patients in each arm was small (127 vs. 134), therefore most of the prediction models did not pass all selection tests and the results have to be interpreted with caution. However, significant models were found for several parameters. Patients receiving TapMono had a better chance to respond in case of a high painDETECT score, little numbness and good mental health status (SF-12 MCS) at randomization. In contrast, patients receiving TapPre had a good chance to respond if they had sleep disturbances, but little anxiety or depression, while the painDETECT score and numbness. In contrast, the painDETECT score and numbness had only little influence in the TapPre group (Table 1). The standardized parameter estimates resulting from the multivariable analyses are shown in Table 2.

**Table 2:** Characterization of parameter estimates for multivariable models.

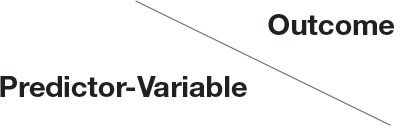	**TapMono**			**TapPre**		
	**MCS^‡^**	**PCS^†^**	**EQ-5D^†^**	**MCS**	**PCS^†^**	**EQ-5D^†^**
HADS A					−0.20	
HADS D				−0.35		
EQ-5D			−0.44			−0.53
painDETECT score		0.38	0.25	−0.16	0.18	
PDQ numbness		−0.27	−0.25			−0.21
SF-12 MCS	−0.47	0.27	0.23	−0.81		
SF-12 PCS		−0.24			0.46	−0.14
Sleep score					0.17	
R^2^adj	0.22	0.57	0.54	0.34	0.45	0.4
R^2^adj for robustness assessment	n.a.	0.10	0.21	0.34	0.14	0.2

*Displayed are standardized parameter estimates for multivariable models, whereby study-inherent variables are not shown. Standardized estimates allow to directly compare the influence of the respective variable on outcome. Adjusted R^2^ is shown for initial model as well as for models regarding possible influence of aberrant data. A decrease of R^2^ indicates missing robustness of the model*.

‡ Coefficient of determination is too low;

† *Failed robustness analysis. Gray hatched: model which passes all predefined criteria. Colors: dark gray p < 0.001; gray p < 0.01; light gray p < 0.05*.

*TapMono, tapentadol PR monotherapy; TapPre, tapentadol PR/pregabalin; MCS, mental component summary scale; PCS, physical component summary scale; EQ-5D European Quality of Life 5 Dimensions; HADS-A, Hospital Anxiety and Depression Scale, subscale anxiety; HADS-D, Hospital Anxiety and Depression Scale, subscale depression; PDQ, painDETECT Questionnaire*.

The model passing all predefined criteria (shaded in hatched gray) indicated that the MCS outcome primarily depends on the MCS value at randomization as well as on HADS D and painDETECT scores. The following equation was calculated:

$$\begin{array}{l} MCS=44.6-\left(0.65*SF12\;MCS\;pre\right)-\left(0.27*PDQ_{Score}\right)\\ \;-\left(0.83*HADS\;D\right) \end{array}$$

**Figure 2:**
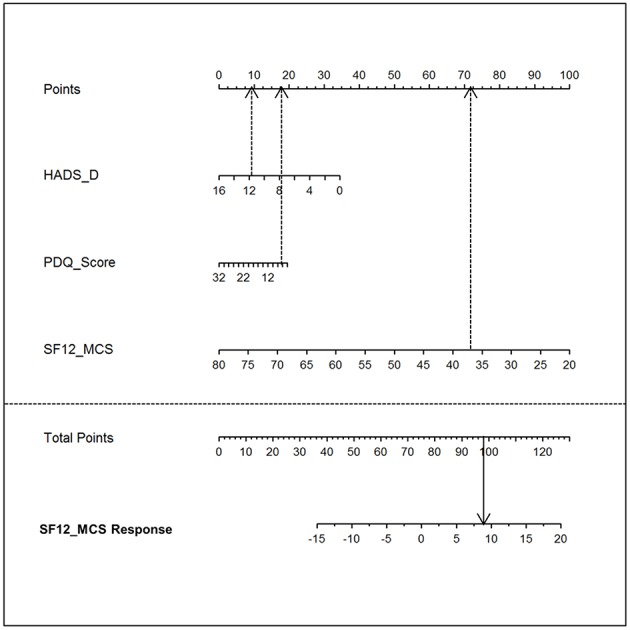
Nomogram. The nomogram visualizes the model equation for the MCS response in the tapentadol PR/pregabalin arm [= TapPre]. To estimate the treatment response, the top line “point” score needs to be determined for each of the three parameters (HADS_D, PDQ_Score, and SF-12_MCS). Thereafter, the sum score (i.e., total points) can be directly assigned to the SF-12_MCS Response (i.e., predicted treatment response). HADS-D, Hospital Anxiety and Depression Scale, subscale depression; PDQ, painDETECT Questionnaire; SF-12_MCS, Short Form 12 Health Survey, mental component summary scale; SF-12_MCS response, Short Form 12 Health Survey, mental component summary scale response.

This equation can also be visualized as a nomogram (Figure 2) which can directly be used to estimate the outcome for a specific patient.

A nomogram visualizes the influence of the different predictive variables on different horizontal lines. Depending on the influence of each predictor, the different lines have different lengths. The longer a horizontal line, the stronger is the influence. The influence of each predictor is determined by projecting the value perpendicularly on the horizontal line at the top resulting in a number of points. By adding the points associated with each predictor resulting in total points, the anticipated magnitude of response can be read on the response horizontal line on the bottom of the nomogram (see Figure 2).

## Discussion

Besides the successful implementation of an objective model for a retrospective identification of predictors for treatment response, several important findings can be summarized:

1. Demographic data (age, BMI, vital signs) are not relevant for response prediction.
2. Patients with a low baseline pain, good sleep quality and high painDETECT score (neuropathic component present) have a good chance to respond very well already to medium doses of tapentadol (qualify for the open-label arm, “optimal responders”).
3. Patients with high painDETECT scores (neuropathic component present), little numbness and a good mental health status after a medium dose tapentadol PR therapy (at randomization) have a better chance to respond to high dose tapentadol PR monotherapy.
4. Patients with sleep disturbances and little anxiety or depression after a medium dose tapentadol PR therapy (at randomization) have a good chance to respond to a combination therapy of tapentadol PR and pregabalin.

The predictor analysis in the study was performed at two different points in time.

First, response criteria were analyzed during the titration period (baseline until randomization, all patients received tapentadol PR 300 mg). Predictors for a satisfactory pain relief (i.e., for “optimal responders”) after the titration period were low pain intensity, good sleep quality, and a high painDETECT score. These results are in line with those of an earlier study with tapentadol in low back pain showing that the intensity of the neuropathic component (high painDETECT score) predicts a good tapentadol outcome (24). However, several caveats of these results should be raised. Patients qualified for this group by an artificial enrichment strategy, i.e., they reach a pain reduction NRS-3 <4 during the treatment with 300 mg tapentadol PR. Thus, as a logical consequence, low baseline pain intensity has to be a predictor for qualification for this group since it is easier to achieve the result with a low baseline pain intensity. However, the other independent co-variables (high PDQ, good sleep) have validity to predict a good response to 300 mg tapentadol PR.

In a second step, the response analysis was performed for the two treatment arms using the co-variables at randomization. This analysis may contribute to a more precise use of the investigated drugs by providing guidance on patients who might benefit from a high-dose monotherapy with tapentadol PR or a combination therapy of a medium dose of tapentadol PR and pregabalin. For the outcome measures “quality-of-life” and “functionality,” the statistical approach identified several significant predictors. In fact, patients with high painDETECT scores, little numbness, and a good mental health status had a better chance to respond to high dose tapentadol PR monotherapy whereas patients with severe sleep disturbances and little anxiety had a good chance to respond to the combination therapy of tapentadol PR and pregabalin. Interestingly, the TapPre group benefitted if a sleep disorder was present which might be due to the well-known positive effect of pregabalin on the sleep architecture.

Since this analysis uses data of a randomized, double-blind controlled study (15), the differential predictive effects in both arms between randomization and endpoint very likely mirror true drug-specific (treatment-dependent) predictive effects. However, although the response model is only valid for a highly selected group of patients (i.e., patients showing a partial response after treatment with 300 mg tapentadol PR), as well as the fact that clinical characteristics at the randomization time point are already diluted by pretreatment with tapentadol, this approach clearly mimics the clinical reality. In clinical practice, patients have already been treated with a medium dose of tapentadol PR as monotherapy and at this time point the clinician has to decide which strategy to choose based on clinical characteristic present at this time.

Our predictor analysis aims to enhance a better understanding of treatment caveats in pain therapy by presenting an approach which helps to assign patients to specific treatment regimens according to objectively determined treatment predictors. However, independently of the identified predictors, the presented study highlights the importance of an appropriate dosage of mono or combination therapies in general. These treatment decisions should be evidence based rather than empirical driven, to achieve the optimum treatment response, especially in accordance with a good tolerability profile.

### Limitations of the Trial

All available characteristics (co-variables) were used to identify potential associations with the endpoints in order to find predictors. Thus, a huge amount of many correlations were calculated (univariable analysis) which will inherently lead to an overestimation of the results. To overcome this bias, several correction steps (model validation) were implemented after having performed a univariable analysis (+ baseline value of outcome variable) and a factor analysis to identify the most relevant independent co-variables. Since the number of patients in each arm was small, many of the prediction models did not pass all selection tests and therefore the results are not very robust. However, these preliminary results can be used to create hypotheses for future research into this important scientific area.

Most of the identified predictors are capable of explaining only 2–10% of the variance of the entire response. Clearly, this is too small to predict the response on a single patient level and the implication to the clinical setting is limited. However, significant predictors that explain only part of the variance might be important to understand new pathophysiological mechanisms that are relevant for prediction. Furthermore, a combination of several independent predictors might be summed up to explain higher levels of the variance and might show relevance even in the clinical setting. Thus, the presented algorithm should be applied to larger numbers of patients in the future. For further improvement of prediction models, the results of the clinical characteristics could be complemented with genetic analysis.

## Data Availability

The datasets generated for this study are available on request to the corresponding author.

## Ethics Statement

The studies involving human participants were reviewed and approved by Ethik-Kommission der Medizinischen Fakultät der CAU zu Kiel, Universitäts-Kinderklinik Arnold-Heller-Straße 3 Haus 9 24105 Kiel. The patients/participants provided their written informed consent to participate in this study.

## Author Contributions

JO and JF drafted the manuscript and configured the tables and figures. JS gave critical input to the manuscript and contributed to the statistical and methodological part. PH provided critical suggestions to the discussion of the manuscript. MH performed the statistical analysis. TK performed the statistical analysis. JG conceived and designed the retrospective analysis. RB designed the analysis and provided critical suggestions to the final version.

### Conflict of Interest Statement

JO reports research support and personal fees from Grünenthal GmbH and travel costs from Pfizer. JF reports grants and personal fees from Grünenthal GmbH, during the conduct of the study; personal fees, and non-financial support from Grünenthal GmbH and Sanofi Genzyme, personal fees from Bayer, non-financial support from Novartis, outside the submitted work. JS reports personal fees from Alnylam Pharamaceuticals and Grünenthal GmbH. PH has received speaking fees from Pfizer and Genzyme and travel reimbursement from Grünenthal. MH is an employee of Statconsult GmbH, Germany. This company reports financial support from Grünenthal GmbH and the University of Kiel. TK has been a contract statistical consultant of Statconsult GmbH, Germany until 12/2018 and reports financial support from Grünenthal GmbH and the University of Kiel. JG has received speaking fees and travel grants from Pfizer, Sanofi Pasteur, and Grünenthal and has been a consultant for Glenmark Pharmaceuticals. RB reports grants/research support from Pfizer, Genzyme GmbH, Grünenthal GmbH, Mundipharma. Member of the EU Project No 633491: DOLORisk. Member of the IMI “Europain” collaboration and industry members of this are: Astra Zeneca, Pfizer, Esteve, UCB-Pharma, Sanofi Aventis, Grünenthal GmbH, Eli Lilly, and Boehringer Ingelheim Pharma GmbH&Co.KG. German Federal Ministry of Education and Research (BMBF): Member of the ERA_NET NEURON/IM-PAIN Project (01EW1503). German Research Network on Neuropathic Pain (01EM0903), NoPain system biology (0316177C). German Research Foundation (DFG); speaker fees from Pfizer, Genzyme GmbH, Grünenthal GmbH, Mundipharma, Sanofi Pasteur, Medtronic Inc. Neuromodulation, Eisai Co.Ltd., Lilly GmbH, Boehringer Ingelheim Pharma GmbH&Co.KG, Astellas, Desitin, Teva Pharma, Bayer-Schering, MSD GmbH, Seqirus, Novartis, TAD Pharma GmbH; he has been a consultant for Pfizer, Genzyme GmbH, Grünenthal GmbH, Mundipharma, Allergan, Sanofi Pasteur, Medtronic, Eisai, Lilly GmbH, Boehringer Ingelheim Pharma GmbH&Co.KG, Astellas, Novartis, Bristol-Myers Squibb, Biogenidec, AstraZeneca, Merck, Abbvie, Daiichi Sankyo, Glenmark Pharmaceuticals, Seqirus, Teva Pharma, Genentech, Galapagos NV, Kyowa Kirin GmbH, Vertex Pharmaceuticals Inc., Biotest AG, Celgene, Desitin, Theranexus.

## Abbreviations

AUC: Area under the curve
BMI: body mass index
CGIC: clinician global impression of change
EuroQol-5D VAS: European Quality of Life 5 Dimensions Visual Analog Scale
EuroQol-5D-Health: European Quality of Life 5 Dimensions
HADS-A: Hospital Anxiety and Depression Scale, subscale for anxiety
HADS-D: Hospital Anxiety and Depression Scale, subscale for depression
NRS: numeric rating scale
PDQ: painDETECT Questionnaire
PGIC: patient global impression of change
ROC: Receiver Operating Characteristic
SF-12 MCS: Short Form 12 Health Survey, mental component summary scale
SF-12 PCS: Short Form 12 Health Survey, physical component summary scale
SQ: Sleep Evaluation Questionnaire
tapentadol PR: tapentadol prolonged release
TapMono: tapentadol prolonged release monotherapy
TapPre: tapentadol prolonged release and pregabalin combination therapy
TRIPOD: Transparent Reporting of a multivariable prediction model for Individual Prognosis or Diagnosis.
